# Primary Focal Segmental Glomerulosclerosis Presenting With Acute Kidney Injury and Nephrotic Syndrome: A Case Report With Sustained Remission

**DOI:** 10.7759/cureus.85499

**Published:** 2025-06-07

**Authors:** Gautam Agrawal, Bhawna Agarwal, Kunal Sonavane, Pallavi Shirsat

**Affiliations:** 1 Nephrology, Independence Health System, Greensburg, USA; 2 Internal Medicine, University of Pittsburgh Medical Center McKeesport Hospital, McKeesport, USA; 3 Department of Internal Medicine/Internal Medicine Hospitalist, Willis Knighton Medical Center, Bossier City, USA; 4 Nephrology, Minden Medical Center, Minden, Louisiana, USA

**Keywords:** acute kidney failure, focal segmental glomerulo-sclerosis (fsgs), heavy proteinuria, hypertension, prednisone treatment

## Abstract

Focal segmental glomerulosclerosis (FSGS) is a common cause of nephrotic syndrome in adults, characterized by segmental scarring of the glomeruli. It can present with proteinuria, hypoalbuminemia, edema, and varying degrees of renal dysfunction. Early diagnosis and treatment are critical to prevent progression to end-stage kidney disease. We report the case of a 53-year-old female who presented with worsening generalized edema, abdominal distension, and significant weight gain. Laboratory workup revealed significant proteinuria, hypoalbuminemia, and acute kidney injury, and kidney biopsy confirmed the diagnosis of FSGS. She was treated with high-dose corticosteroids, followed by initiation of losartan, and over a two-year follow-up period, she achieved and maintained complete remission with stable renal function and no recurrence of proteinuria. This case highlights the importance of prompt diagnosis and early intervention in FSGS. High-dose corticosteroids remain the cornerstone of treatment for primary FSGS, with adjunctive renin-angiotensin system blockade playing a key supportive role. Long-term follow-up is essential to monitor treatment response and maintain remission.

## Introduction

Focal segmental glomerulosclerosis (FSGS) is a group of clinical and pathological syndromes characterized by a distinctive glomerular lesion. This lesion arises from various external or intrinsic factors that impair podocyte function [1]. FSGS is defined by segmental sclerosis affecting some (focal) glomeruli and only parts of the affected glomeruli (segmental), as observed on kidney biopsy. It is a leading cause of nephrotic syndrome in adults, accounting for approximately 40% of cases in the United States [1], and is the most common primary glomerular disease among patients with end-stage renal disease (ESRD) in the U.S. [2]. Black individuals face a fourfold increased risk of developing FSGS-related ESRD compared to White individuals and tend to progress to ESRD at a younger age [2]. FSGS can be classified as either primary (idiopathic) or secondary, due to conditions such as hypertension, obesity, viral infections, exposure to nephrotoxic drugs, or genetic mutations [1,3].

Clinically, FSGS typically presents with proteinuria (nephrotic-range in 50-60% of adults), hypoalbuminemia, edema, and varying degrees of renal dysfunction. Diagnosis is confirmed via kidney biopsy, which reveals focal and segmental sclerosis on light microscopy and widespread podocyte foot process effacement on electron microscopy [3].

This case report describes a 53-year-old female who presented with significant proteinuria and acute kidney injury, ultimately diagnosed with primary FSGS. The case underscores the importance of a comprehensive diagnostic evaluation, early initiation of immunosuppressive therapy, and long-term follow-up to achieve and maintain remission.

## Case presentation

A 53-year-old White woman presented to the hospital with complaints of worsening edema, abdominal distension, and a 21-pound weight gain over the preceding two weeks. She also reported decreased urine output but denied foamy urine, gross hematuria, upper respiratory symptoms, or gastrointestinal complaints. Her past medical history was significant for hypertension, gastroesophageal reflux disease, and hyperlipidemia.

On physical examination, she was awake, alert, and oriented, and in no acute distress. Her lungs were clear to auscultation bilaterally without wheezes or crackles. Heart sounds were regular. She appeared hypervolemic with 2+ lower extremity edema and generalized anasarca. A malar rash was noted over her cheeks. Initial laboratory results were notable for a serum creatinine of 1.7 mg/dL (baseline creatinine was 0.9 mg/dL three months prior) and a blood urea nitrogen (BUN) of 55 mg/dL, as shown in Table 1. Urinalysis showed 600 mg/dL of proteinuria, and serum albumin was 3 g/dL. Autoimmune serologies, including antinuclear antibody (ANA), anti-double-stranded DNA, and anti-neutrophil cytoplasmic antibodies (ANCA), were negative. Complement levels (C3, C4) were within normal limits. The hepatitis panel and HIV test were negative.

**Table 1:** Laboratory Analysis ANA: antinuclear antibody; anti-dsDNA: anti-double-stranded DNA; LDL: low-density lipoprotein; HIV: human immunodeficiency virus

Laboratory Tests	Results	Reference Range
Serum Albumin	3.0	3.5-5.7 g/dL
Blood Urea Nitrogen (BUN)	55	7-25 mg/dL
Creatinine	1.7	0.6-1.2 mg/dL
Sodium	138	136-145 meq/L
Calcium	8.4	8.6 - 10.3 mg/dL
Hemoglobin	13.6	11.7-15.8 gm/dL
Cholesterol	209	< 200 mg/dL
LDL	122	< 101 mg/dL
ANA	0.19	<0.8-Negative
Anti-ds DNA	Negative	<25-Negative
Hemoglobin A1c	5.7	5.7-6.4 (Prediabetic) %
Hepatitis B Surface Ag	Non-reactive	Non-reactive
Hepatitis C Ab	Non-reactive	Non-reactive
HIV	Non-reactive	Non-reactive

**Figure 1:**
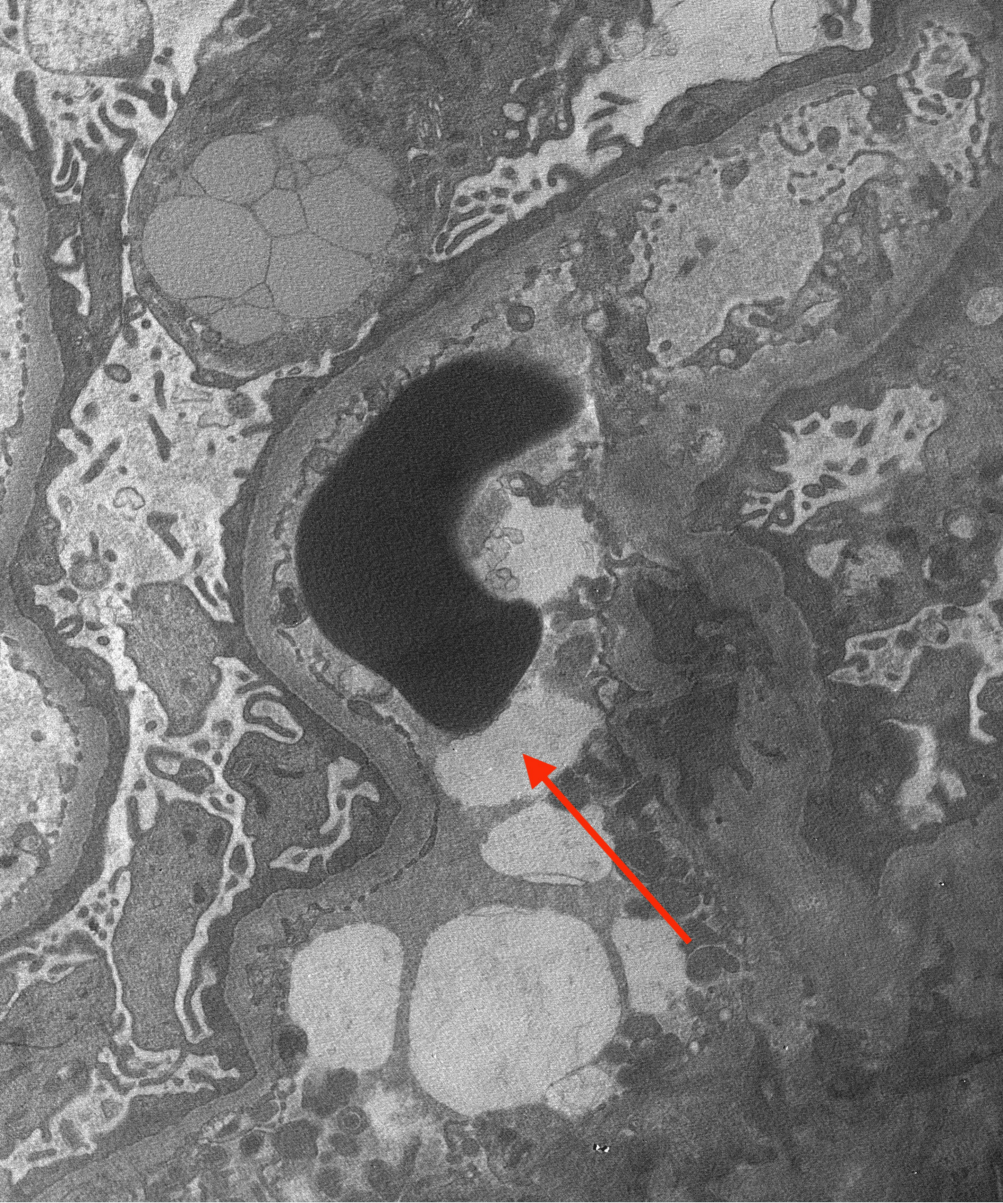
Electron microscopy (EM) image of kidney biopsy Red arrow showing podocyte foot fusion effacement.

The major differential diagnosis in her case was lupus nephritis, given her severe acute kidney injury and malar rash. A kidney biopsy was performed, which revealed FSGS with ultrastructural features consistent with primary podocytopathy as shown in Figure 1. Additional findings included mild interstitial fibrosis and tubular atrophy, as well as mild arterial and arteriolar hyalinosis and sclerosis.

She was started on prednisone 60 mg daily and intravenous diuretics, which led to symptomatic improvement. However, her renal function initially worsened, with creatinine peaking at 3.6 mg/dL before gradually improving with corticosteroid therapy. At the time of discharge, her creatinine had decreased to 1.5 mg/dL. She was initiated on losartan, which was gradually up-titrated. Follow-up was arranged in the nephrology clinic. She tolerated steroids well, which were tapered slowly over a period of four months. Over the subsequent two years, the patient has been closely followed in the nephrology clinic and is doing very well. Her renal function has remained stable, with a creatinine of 0.8 mg/dL and no evidence of proteinuria on urinalysis. Her blood pressure has been well controlled with losartan.

## Discussion

FSGS is not a single disease entity but rather a manifestation of diverse etiologies, including primary (presumed immunologic), secondary (obesity, viral, drug-induced), and genetic forms, each with distinct clinical presentations and therapeutic responses [3,4]. Our patient presented with significant proteinuria, hypoalbuminemia, generalized edema, and acute kidney injury (AKI). These findings, in the absence of an identifiable secondary cause or autoimmune etiology, supported a working diagnosis of primary FSGS. Although she had a history of hypertension, it was well controlled, and there were no other contributing comorbidities such as diabetes, obesity, or known drug exposures. The presence of a malar rash raised concern for systemic lupus erythematosus (SLE); however, her serological workup, including ANA, dsDNA, and complement levels, was unremarkable, effectively ruling out lupus nephritis.

Diagnosis was confirmed by kidney biopsy, which remains the gold standard for FSGS classification. Therapeutic strategies are tailored to the underlying etiology. For primary FSGS, immunosuppression with corticosteroids remains the mainstay for treatment. Calcineurin inhibitors or rituximab are alternatives in steroid-resistant or steroid-dependent cases [5,6]. Treatment options like adalimumab or abatacept have also shown potential therapeutic benefit but still require larger studies to determine their efficacy and safety [6]. Therapeutic response rates and long-term outcomes are variable, and response to therapy is an important prognostic factor. Our patient was started on prednisone 60 mg daily. Although her renal function initially worsened, likely due to her underlying disease activity and fluid overload, she showed gradual and sustained improvement with corticosteroid therapy. Her serum creatinine improved from a peak of 3.6 mg/dL to 0.8 mg/dL over two years of follow-up. Renin-angiotensin-aldosterone system (RAAS) inhibition with angiotensin-converting enzyme (ACE) inhibitors or angiotensin receptor blockers (ARBs) has a key role in treatment to reduce proteinuria and slow renal disease progression [7]. The addition of losartan to our patient contributed to blood pressure control and the reduction of proteinuria.

Secondary and genetic forms generally do not benefit from immunosuppression and are managed with supportive measures like RAAS inhibition and controlling the underlying etiology. Recent clinical trials on sparsentan, a dual endothelin-angiotensin receptor antagonist, have demonstrated promising antiproteinuric effects, suggesting a potential role in the future management of FSGS. However, the lack of significant difference in eGFR decline over two years means that its long-term impact on kidney function remains uncertain [8].

Complete remission, defined by resolution of proteinuria and renal function stabilization, was achieved and maintained in this case. Favorable prognostic factors include initial responsiveness to steroids, absence of significant chronic changes on biopsy, and early initiation of treatment [7]. This outcome emphasizes the importance of early diagnosis, appropriate immunosuppressive therapy, and long-term monitoring.

## Conclusions

This case highlights the clinical presentation, diagnostic workup, and therapeutic approach to primary FSGS in a patient who presented with nephrotic-range proteinuria and acute kidney injury - an atypical presentation, as many cases of FSGS occur with stable renal function. The presence of a malar rash initially raised concern for lupus nephritis, but kidney biopsy confirmed FSGS with features of primary podocytopathy. Despite the generally variable or poor steroid response seen in adults with FSGS, this patient achieved complete remission with corticosteroids alone, without additional immunosuppression, and has remained in remission for over two years. This underscores the importance of early biopsy, accurate diagnosis, and prompt initiation of therapy. Adjunctive treatment with RAAS inhibition supported long-term renal stability and blood pressure control. The case also reinforces the need for individualized care and highlights the evolving role of molecular and genetic insights in guiding the management of this complex glomerular disease.
